# Recent advances in managing overactive bladder

**DOI:** 10.12688/f1000research.26607.1

**Published:** 2020-09-11

**Authors:** George Araklitis, Georgina Baines, Ana Sofia da Silva, Dudley Robinson, Linda Cardozo

**Affiliations:** 1 Department of Urogynaecology, King’s College Hospital, London, UK

**Keywords:** Advances, overactive bladder, OAB, treatment

## Abstract

Overactive bladder syndrome (OAB) is defined as urinary urgency, usually accompanied by frequency and nocturia, with or without urgency incontinence, in the absence of urinary tract infection or other obvious pathology. In this review, we focus on recent advances in the management of OAB. We examine the evidence on the effect of anticholinergic load on OAB patients. Advances in medical treatment include a new beta-3 agonist, vibegron, which is thought to have fewer drug interactions than mirabegron. Treatment of genitourinary syndrome of the menopause with oestrogens and ospemifene have also shown promise for OAB. Botulinum toxin has been shown to be an effective treatment option. We discuss the new implantable neuromodulators that are on the market as well as selective bladder denervation and laser technology.

## Introduction

Overactive bladder syndrome (OAB) is defined as urinary urgency, usually accompanied by frequency and nocturia, with or without urgency incontinence, in the absence of urinary tract infection or other obvious pathology ^1^. The prevalence quoted in studies varies between 1 and 38.8% ^2^. OAB is projected to cost the United States $82.6 billion in 2020 ^3^. It can have a negative impact on health-related quality of life and work productivity ^4^. Those with bothersome symptoms have higher levels of anxiety and depression ^4^. Treatment of OAB includes fluid management, behaviour modification, drug therapy, neuromodulation, and, rarely, surgery. In this review, we focus on recent advances in managing OAB.

## Anticholinergic load

Anticholinergic load is the cumulative effect of taking medication with anticholinergic properties ^5^. Many elderly people take numerous medications for various chronic medical conditions, including urinary incontinence, that exert anticholinergic properties ^5^. A high anticholinergic load can lead to physical and cognitive impairment in older adults ^6^.

An observational retrospective cohort study of 113,311 patients aged over 65 years investigated falls, fractures, and mortality in those taking anticholinergics for OAB compared to those unexposed to anticholinergics ^7^. There was a 1.28-fold increased risk of falls and fractures in individuals taking anticholinergics compared to controls. Past exposure to anticholinergics for OAB resulted in an increased risk of 1.14 for falls and fractures compared to controls.

A UK case controlled study of 40,770 adults aged over 65 years from GP data investigated the association between anticholinergics and dementia ^8^. Drugs used for OAB were significantly associated with dementia. When the drug was used 4–10 years, 10–15 years, and 15–20 years prior to dementia diagnosis, there were odds ratios (ORs) of 1.23, 1.22, and 1.27 ( *P* <0.01), respectively. The drugs predominantly used were oxybutynin and tolterodine, which are both older, non-selective, generic medications used to treat OAB.

A US observational study investigated 350 patients aged 65 years or older over 3.2 years ^9^. Use of anticholinergic medication was associated with an increased risk of transition from normal cognition to mild impairment (OR 1.15, *P* = 0.03). A prospective cohort study of 3,434 patients using anticholinergics found that 23% developed dementia over a mean of 7.3 years ^10^. Former users of these drugs had similar risks compared to recent or continued use. Using oxybutynin for over 3 years had the greatest risk of dementia.

A systematic review found 15 out of 27 studies showed a positive correlation between anticholinergic load and mortality ^6^. Ten studies found no correlation, and two studies showed mixed results, which may be related to follow up and the quality of the studies. The studies that showed a positive correlation were mainly of high quality.

With these effects in mind, care should be taken when prescribing OAB medication for older adults. Management of these patients should address mobility, functional impairment, lifestyle modification, urgency suppression, bladder retraining, patient and caregiver expectations, and life expectancy ^11^. Despite this, drug therapy may be needed if incontinence does not improve with these supportive measures. Trospium chloride is a quaternary amine anticholinergic, compared to the others, which are tertiary amines. This means trospium chloride is theoretically less likely to cross the blood–brain barrier, and this reduces central nervous system side effects ^12^. A randomised double-blind placebo-controlled trial of women aged over 50 years with OAB were treated with placebo or trospium chloride ^13^. There was no significant difference in scores of cognitive tests between placebo and treatment at week 4.

Alternatively, mirabegron could be used as a treatment in this population. A phase IV study found that mirabegron improved symptoms of OAB in the elderly, with safety and tolerability consistent with known mirabegron safety profile ^14^. A subanalysis of the BESIDE study in those aged over 65 years, which investigated the combination of solifenacin and mirabegron, found no difference between solifenacin 5 mg plus mirabegron 50 mg compared to solifenacin 10 mg ^15^. The authors argue that combination therapy may be better for the elderly rather than solifenacin 10 mg, as the latter may increase the anticholinergic load. Another study found that there was no significant change in the Montreal Cognitive Assessment from baseline to week 12 in elderly patients using mirabegron ^16^.

## Advances in medical treatment

### Beta-3 agonists

Two classes of drug therapy are employed in the management of OAB. There have been no recent advances in anticholinergic medication, which is the main pharmacological treatment for OAB. Mirabegron, a beta-3 agonist, has been used as an alternative to anticholinergics since 2013 ^17^. In September 2018, vibegron, a selective beta-3 adrenoreceptor agonist, was approved in Japan for the treatment of OAB ^18^. It is unlikely to be metabolised by CYP3A4 or CYP2D6 ^19^; therefore, there is a low risk of drug interactions. Mirabegron inhibits CYP2D6 and is therefore a source of drug interactions ^20^.

A phase III multicentre prospective study investigated vibegron in an OAB population over 12 months ^21^. There were significant improvements in OAB symptoms from baseline compared to week 4 and continued improvement until week 52. There was also a significant improvement in all King’s Health Questionnaire (KHQ) score domains, except for general health perception. The KHQ is a disease-specific quality of life questionnaire for patients with urinary incontinence.

A multicentre, randomised, four-arm, parallel-group, placebo-controlled phase III study of patients with OAB was performed on 1,230 patients ^22^. There were significant improvements in OAB symptoms with vibegron over placebo. There were also improvements in all KHQ score domains, except for general health perception and personal relationships. Adverse events were similar amongst placebo, vibegron, and imidafenacin (an anticholinergic) groups. There were no changes in vital signs.

### Phosphodiesterase type 5 inhibitors

Phosphodiesterase type 5 inhibitors (PDE5Is) are used to treat erectile dysfunction. A randomised, double-blind, placebo-controlled trial investigated the efficacy and safety of daily low-dose tadalafil for 96 female patients with OAB ^23^. There were significant improvements in the overactive bladder symptom score and Indevus Urgency Severity Scale. There were significant reductions in OAB symptoms compared with baseline and placebo. There were no reported serious adverse events. It is hypothesised that tadalafil reduces the contraction of the detrusor muscle. Further larger and longer studies are needed to confirm these results. At present, these medications are not considered first-line therapy for OAB.

## Genitourinary syndrome of menopause treatment for OAB

Genitourinary syndrome of menopause (GSM) is the new terminology for vulvovaginal atrophy and urinary symptoms, which may occur together or independently ^24^. There is evidence to show that oestrogen deficiency may increase the risk of OAB ^25^. A meta-analysis reviewed 10 randomised placebo-controlled trials ^26^. It found that oestrogen therapy was superior to placebo for frequency, nocturia, and urgency incontinence. Vaginal oestrogens were better than systemic oestrogens for urinary urgency.

Combination therapy in the form of vaginal oestrogens and anticholinergics may help improve OAB symptoms. One study investigated solifenacin, with or without intra-vaginal promestriene, in 104 postmenopausal women over 12 weeks ^27^. Both groups had a significant reduction in frequency, urgency, urgency incontinence episodes, and nocturia but without a significant difference between the groups. The combination group did have a significantly better reduction in OAB symptom score questionnaire ( *P* = 0.016) compared to solifenacin alone.

Another study investigated fesoterodine with or without vaginal premarin ^28^. Both groups had improvements in OAB symptoms. The combination group had better improvement in OAB symptom score questionnaire, OAB health-related quality of life score, and sexual quality of life questionnaire scores.

Ospemifene is a selective oestrogen receptor modulator that has an oestrogen agonist effect and can be used by those in whom oestrogen therapy may be contraindicated ^29^. A study of 105 patients found a significant improvement in frequency, nocturia, urgency, and urgency incontinence ^29^. There were also significant improvements in OAB questionnaire scores. A retrospective review of 46 women using ospemifene for GSM found a significant improvement in frequency, nocturia, urgency, and urgency incontinence ^30^.

## Onabotulinumtoxin A

OnabotulinumtoxinA (“botox”) helps those with OAB by inhibiting acetylcholine release, leading to bladder detrusor muscle paralysis ^31^. A systematic review and meta-analysis has shown significant incontinence-free episodes ( *P* <0.001), reduction in urinary incontinence episodes ( *P* <0.001), and reduction in mean number of micturitions ( *P* <0.001) ^32^.

A recent meta-analysis of 19 studies found that doses of 200 U and 300 U were more effective than placebo for the treatment of neurogenic detrusor overactivity, with minimal but manageable side effects ^33^. A double-blind, randomised, placebo-controlled trial investigated different doses of botulinum toxin ^34^. A dose greater than 150 U had minimal additional improvement in symptoms, with increased risk of urinary retention and need to self-catheterise. The authors recommend using 100 U, which they feel balances symptom improvement and safety profile. The National Institute for Health and Care Excellence (NICE), which provides health guidance in the United Kingdom, recommends using 100 U ^35^.

A recent study investigated bladder trigone-involved injections against those with trigone-sparing injections ^36^. A total of 103 patients received 100 U and were followed up at 6 months. Both groups had a reduction in OAB outcome measures, but there was no difference between groups. The trigone-involved group had a higher incidence of urinary tract infection and voiding difficulties.

## Neuromodulation

### Posterior tibial nerve stimulation

Posterior tibial nerve stimulation involves the use of electrical impulses and is a form of neuromodulation employed to improve urinary symptoms ^37^. This can be in the form of percutaneous tibial nerve stimulation (PTNS), which uses a needle, and transcutaneous tibial nerve stimulation (TTNS), which uses pads. This treatment usually involves weekly 30-minute visits to the hospital for up to 12 weeks. More recently, implantable devices have become available.

The RENOVA iStim™ is a wirelessly powered implant. The implant is surgically inserted under local anaesthesia. The incision is made 3 cm superior and 2 cm posterior to the medial malleolus. The electrode is placed near the tibial nerve and secured with a non-absorbable suture. An external control unit is worn around the ankle to activate the electrodes. This unit is worn six times a week for 30 minutes to activate treatment ^37, 38^. A study of 15 patients (13 female) showed a significant improvement in frequency, urgency, and urgency incontinence episodes at 3 months ^38^. There was also significant improvement in quality of life. Three patients required a week of antibiotics and three required a week of analgesics. One device was explanted for suspected infection, although cultures were negative. None of the patients reported difficulty in operating the device.

A 6-month study evaluated the implant in 36 patients ^39^. A total of 71% of patients experienced clinical success (>50% reduction) at 6 months. The number of leaks per day, leak severity, frequency, degree of urgency, and pad changes per day reduced significantly. A total of 28% of patients with urgency incontinence were dry. There was significant improvement in OAB-q questionnaire scores. Adverse events were noted in 47% of patients; 14% had pain, 22% suspected infection, and 8% wound complications. One patient had the implant removed owing to pain and swelling, but cultures excluded infection. A total of 20 patients from the previous study were enrolled in a 3-year follow up study ^40^. A total of 75% had a >50% improvement in symptoms, with significant improvement in quality of life. The majority of patients were moderately or very satisfied. No adverse events were reported between 6 months and 3 years.

Another implant is the primary battery-powered, nickel-sized and -shaped neuromodulation device called the eCoin® for tibial nerve stimulation to treat refractory urgency urinary incontinence. A study of 46 patients found a significant reduction in urgency incontinence episodes ^41^. A total of 72% had an improvement in symptoms, with 20% dry at 6 months. One adverse event was recorded secondary to infection, which was successfully treated with intravenous antibiotics. This device is still undergoing FDA clinical trials ^42^.

### Sacral neuromodulation

In 1997, the FDA approved sacral neuromodulation (SNM) for the treatment of refractory urgency, urgency urinary incontinence, and frequency ^43^. It has been implanted in over 200,000 patients, with success rates between 62 and 90% ^43^. It does carry frequent adverse events, with surgical revision between 3 and 16% ^43^. The device will need to be replaced after the battery runs its lifespan, with replacements occurring on average after 62.5 months ^44^. The manufacturer of the InterStim device, Medtronic, recommends that MRI should not be performed in patients with the device ^45^. They allow MRI of the head only if the device is turned off and a 1.5-Tesla magnet or lower is used.

Recently, an implantable rechargeable SNM system by Axonics has come on the market. This has addressed the issue of the shorter battery lifespan of the InterStim with a rechargeable lithium ion battery, lasting up to 15 years or longer ^46^. With typical use, the battery should last 2 weeks before needing to be recharged. This is done by a wireless charger and takes 1 to 2 hours ^47^. It was designed so that the implantation procedure was nearly identical to the InterStim ^47^. The Axonics SNM is 60% smaller than the InterStim, with the goal of reducing the risk of discomfort and making it more suitable for those with a lower BMI ^47^. It is made of titanium and ceramic rather than just titanium. The ceramic has a ferrite core, which reduces heat generation during charging and allows charging of up to 3 cm depth ^46^. It also relies on a current controlled system rather than the voltage controlled system of the InterStim. Therefore, if tissue resistance increases with time, the voltage will increase automatically to maintain constant current to the nerve, avoiding the need to manually increase the current ^47^. The Axonics rechargeable neurostimulator is safe for MRI with 1.5-Telsa full body and 3-Tesla head MRI ^48^.

The rechargeable device is thought to save $27,121 per patient over 15 years ^49^. It was projected it could save the United States healthcare system up to $12 billion. NICE recommends the use of the Axonics SNM. It estimates cost savings of £6,200 per person, which begin 6 years after implantation ^50^. The expected publication of the NICE guideline was June 2020.

A prospective, multicentre study implanted 51 patients suffering from OAB with the rechargeable SNM device ^51^. At 12 months, 96% experienced a reduction in urinary incontinence ( *P* <0.001) and 71% had a significant reduction in urinary frequency ( *P* <0.001). There was a significant improvement in quality of life ( *P* <0.001). At 1 year, 77% of all subjects were very or moderately satisfied and 79% would recommend the treatment to a friend. The duration of charging was acceptable to 98%, with 83% finding it easy to charge. Adverse events occurred in 25% of subjects. The most common was undesirable or uncomfortable stimulation, which was resolved with reprogramming. Pain at the implant site occurred in 2%. There was one incident of lead migration. One subject had an infection requiring explantation 3 weeks later. Two other patients had their device removed because of lack of efficacy.

If SNM fails, treatment of refractory OAB can be very difficult. A study of 52 patients in whom SNM did not work found botulinum toxin to be a successful treatment in 27% of cases ^52^. A recent study assessing the cost effectiveness of SNM or 200 U botulinum toxin for the management of refractory OAB found the former to incur higher costs ^53^. At 2 years, SNM cost $35,680 compared to $7,460 for botulinum toxin and at 5 years cost $36,550 compared to $12,020. There was no difference in reduction in urgency incontinence episodes between the treatments at 2 years.

## Selective bladder denervation

Selective bladder denervation (SBD) is a procedure that involves radiofrequency ablation of the sub-trigone area of the bladder, which contains afferent sensory nerves ^54^. The device is applied to the trigone under cystoscopic guidance. The thermal delivery probe is placed along the left border of the trigone 5 mm below the ureteric orifice. The electrodes are then advanced 3 mm into the urothelium and ablation begins. This is repeated on the right border of the trigone and again in numerous points in between these two borders.

A total of 63 patients with refractory OAB underwent this treatment ^54^. At 12 weeks, there were significant reductions in frequency, urgency episodes, and urgency incontinence episodes. There was a significant improvement in OAB questionnaire scores and quality of life ( *P* <0.01). Ablation for 60 seconds had significantly greater improvement in urgency incontinence episodes and quality of life scores than ablation for 10 seconds, with no significant difference in post void residuals. Post procedure pain was minimal over 5 days. One woman had a serious adverse event of obstruction of the left ureter, leading to hydronephrosis and pyelonephritis 8 days later. Lower urinary tract infection occurred in 9.5%. No patients had urinary retention requiring catheterisation.

This study was performed under sedation or a general anaesthetic. The authors are planning to study the use of local anaesthesia. This human feasibility study was also non-randomised, which introduces bias. The results and safety profile are promising, but larger controlled studies with longer follow up are needed.

## Laser treatment

Light amplification by stimulated emission of radiation (laser) was first described by Einstein in 1917 ^55^. It is used for different medical reasons. The two most commonly used lasers are microablative fractional CO _2_ laser (SmartXide ^2^-V ^2^LR, MonaLisa Touch; DEKA, Florence, Italy) and non-ablative photothermal erbium:YAG (Er:YAG) laser (Fotona Smooth XS; Fotona, Ljubljana Slovenia) ^55^. The laser causes thermomodulation by heating and (in the case of the CO _2_ laser) ablating columns of tissue ^55^.

A prospective observational pilot study ^56^ enrolled 30 postmenopausal women with vulvovaginal atrophy and OAB. They were treated with CO _2_ laser, with three treatments 30 days apart. The procedure was performed in an ambulatory setting without analgesia or anaesthetic. There were significant improvements in bladder diary values ( *P* <0.0001), number of urgency episodes ( *P* <0.0001), urgency incontinence episodes ( *P* = 0.006), and OAB questionnaire scores ( *P* <0.0001). There were no adverse events.

Another study recruited 150 postmenopausal patients to Er:YAG laser (three treatments, 1 month apart), an anticholinergic (fesoterodine), or mirabegron ^57^. There were significant improvements in OAB symptom scores in all three groups ( *P* <0.001), but only the laser group reported improvement in vaginal health index scale at 12 months. There were no adverse events with the laser. A further study of 30 women completed two sessions of Er:YAG laser treatment ^58^. There were significant improvements in OAB symptom scores at 3 months ( *P* = 0.027) but not at 12 months ( *P* = 0.576). No major adverse events occurred. Most described mild pain and few had vaginal discharge or spotting for several days.

At present, there is little evidence to support the use of laser for OAB. Further larger controlled studies are needed.

## Conclusion

This article highlights the recent advances in the management of OAB. When prescribing for older adults, care is needed because of the risks associated with a high anticholinergic load. Alternative treatments may be needed, including mirabegron, which does not add to the load. Vibegron is a promising beta-3 agonist that has potentially fewer drug interactions but is licensed only in Japan. Further studies are needed for PDE5Is. Botulinum toxin has been shown to be a good treatment option. There have been promising developments in implantable devices for both PTNS and SNM, with the latter rechargeable device being implemented soon in the UK. SBD and laser are new technologies that show improvements in OAB, but further studies are needed before they can be recommended as treatments.
